# Screw Worms in the Nose

**Published:** 1891-12

**Authors:** T. C. Osborn

**Affiliations:** Cleburne, Texas


### SCREW WORMS.

REPLY TO DR. GILBERT'S REQUEST IN NOVEMBER NUMBER.

CLEBURNE, TEXAS, Dec. 14, 1891.

*Editor Daniel's Texas Medical Journal;*

It is quiet a pleasure, as well as a duty, my replying to Dr. Gilbert, giving my observation in the treatment of screw worms in the nose, and for "itch in horses." I make no doubt the doctor means the human nose, but if I should be wrong it makes no difference, as the treament is applicable alike to man or beast. In many instances of screw worm troubles since my temporary stop in Louisiana, and my residence in Texas, where these pests are very common, until lately, I resorted to the application of calomel alone, to the affected parts, and never had occasion to make it more than once. All the preparations of mercury are equally applicable, but this one gives all the satisfaction desired and is handy everywhere. It is alike destructive to all the maggot family.

Our farmers here use it with cattle, first using chloroform, pouring it freely over the ulcerated surface, and then following it with calomel covering the part. As far as I can judge the chloroform alone is amply sufficient, as it destroys the worm at once, as may be seen by the *frothing* when applied. It is also a valuable remedy in the itch of horses, especially if followed up by the liberal use of peroxide of hydrogen well rubbed on.

Of late I find the peroxide—Marchand's—alone, very effectual in the human nose, either for nasal catarrh or the screw worm, and like chloroform, the frothing is the best evidence of its destructive action on the ulcerous surface, with or without the presence of worms.

I am now treating all my cases of nasal catarrhs with it, and find the spray quite effectual in bringing about a healthy condition of the cavities. My usual advice is, "continue the spray two or three times a day as long as it froths." In the healthy surface there is no froth.

It is hardly necessary, I deem it, to remind the operator, that the spray instrument must have no metal in its construction; rubber only, and liberal vents at the point. Do not fear it, it is harmless to healthy surfaces, and manifesting only a gentle heat to the ulcerous parts. My conviction is that peroxide of hydrogen is the best of all dressings for ulcers of any kind.

